# Self-Styled Doctors

**Published:** 1870-04

**Authors:** 


### SELF-STYLED DOCTORS.

It would seem that when a rogue has reached his wit's end in his endeavor to fleece the public, and has failed, that he immediately commences business as a traveling doctor, and in that he is sure to succeed.

Recently our citizens were astonished at the liberality of one of these traveling concerns, who engaged Stancliff Hall for a free lecture, in which he proclaimed that he was prepared to heal any sort of a disease for the small sum of three dollars. If no cure was secured to the patient, no pay would be demanded. Of course, all classes of people flocked to the rooms of this wondrously benevolent gentleman, until the halls and parlors at the Rathbun were overrun with invalids, seeking the presence of this great healer. The circular distributed to those in waiting, announced the wonderful doctor to be "Prof. D. Wilson, of the Rochester Infirmary." The Professor also had some wonderful chap with him who did the lecturing(?) and whose Frenchy name has now escaped us. However, they proceeded with their victims in the following novel and original manner, to wit: The patient is informed that his disease is curable; (of course) that, in order to perform this cure, medicine is necessary; (quite likely) and that this medicine will cost the patient the very small sum of three dollars, but that it will be immediately refunded if it does not cure. The Professor then takes the three dollars and furnishes his confiding victim with the following blank, which is a marvel of ingenuity and shrewdness:—

"I, M......... do solemnly swear that I have used all the prescribed medicines three times every day for three months, and have in all things followed the prescriptions and directions furnished by Dr. Wilson, and am not cured.

..................................

Sworn to and subscribed before me this..............day of...............1869. .....................Justice of Peace.

N. B.—Forward this by mail (stamp enclosed) to Prof. D. Wilson, Rochester Infirmary, Rochester, N. Y., and you will receive your money by return mail."

It is here stipulated with the sickly one that, if he uses the "prescribed medicines three times a day for three months," and is no better, his money (three dollars) will be returned to him. After the Professor had left the city, the patient discovered that in order for him to carry out his part of the agreement, it becomes necessary for him to purchase a bottle of medicine about once a week, for three months, at $3.00 a bottle, of some druggist in the city mean enough to accept such an agency; and he must expend at least *thirty-six dollars* in so doing; and *then* if he finds himself no better, he can claim his *three dollars* by filling up the above blank and forwarding the same to "Prof. D. Wilson, of the Rochester Infirmary"!! Now, isn't that cute? The dear benevolent soul! How kind he has been to thousands of the poor, sickly ones throughout the land. "Let us kiss him for his mother!" It will at once be apparent to the more practical observer that the Professor has made a clear gain of about *thirty-three dollars* on the transaction, provided you have been stupid enough to follow up his "treatment;" and, if you have not obeyed orders, it is plain enough to be seen that he has made a very handsome profit on his little bottle of medicated water, while you have been assisting him in doing a very thriving and remunerative business.

So much for the mode by which this gay and festive Professor prosecutes his business. Now, let us inquire into his antecedents—let us ascertain how he came by his medical knowledge, so that the dear public may know what sort of a "doctor" they have been employing. Don't be shocked, dear reader, when we tell you that this Professor never read medicine an hour in his life; that he knows nothing under the sun of medicine or disease; that he can't frame a grammatical sentence, of a dozen words, to save his life; that he is simply a broken down theatrical manager, whose "show" was seized to satisfy hall and hotel claims, and that he was forced into his present business because it promised better success than that of running a fourth-rate theatre through our smaller villages.

The Professor has appeared before the public in a multiplicity of characters during the past five years. He was "Professor King" in 1865, and was doing sleight-of-hand performances. Soon after he appeared with "Wilson's New Orleans Minstrels," as the "Great American Sampson," and amused the people by his feats of balancing feathers, whips and chairs on his nose. In 1866-7 he was known as the "Celebrated Indian Physician from beyond the Rocky Mountains," and sold a wonderful coughing compound which he dubbed "What is it!" Next, we learned of him as one of the proprietors of "Henry, Carnier & Wilson's Broadway Theatre." This went to smash, after which he came to this city and exhibited in Concert Hall, in 1868, as "Wilson's Broadway Theatre." In the evening he did the light Irish characters, for the Theatre, and during the day was known at the Brainard House, as the "Great Indian-Tape-Worm-Doctor." *Now* we know him as "Prof. Wilson, of the Rochester Infirmary." Isn't that a delightful record for a physician? Do'nt you think he ought to receive abundant patronage from the afflicted? How many of our readers are willing to trust themselves in the hands of such men? Yet, this is the very risk they run every time they patronize one of these abominable traveling institutions.

We desire now to direct your attention to another individual who has been palming himself upon the citizens of this place for a physician, and who has done quite a successful business in this vicinity. We have his history from several reliable gentlemen, and it runs as follows:

Dr. Gleason, of Philadelphia, a noted physiological lecturer, who is widely and favorably known throughout the country, keeps constantly employed, a bill poster, whose duty it is to see that the bills announcing the lectures are duly posted, halls engaged, tickets taken at the door, and circulars, announcing the next lecture, handed to the audience as they retire from the hall, &c. Two years ago the Doctor engaged two brothers, by the name of STEVENS, to do this work. One went in advance of the lecturer to do the bill posting, while the other remained with the Doctor, to sell tickets and to make himself generally useful about the hall. The Doctor, being a good lecturer and a well-read physician, and his door keeper being constantly with him, it was not surprising that the lackey should take in some of the Doctor's knowledge by absorption,—as it were—of course not! Therefore, have we a celebrated doctor, who was formerly physician to the "Crystal Springs House," in Yates County, but more recently of the Rathbun house, in this city. Now, this gentleman *may be* an excellent physician for aught we know—but the manner in which he became a physician does not accord with our ideas of a medical education—that's all. Of course, those who feel disposed to employ such a man to treat disease, can do so,—it is simply none of our business.

This article, possibly, will create something of a sensation,—it is intended that it should—as both these self-styled-doctors have been doing quite a lucrative business in this vicinity, but we desire it to be distinctly understood that we do not write this out of malice, for they have done us no harm, neither have we ever seen either of them that we are aware of. We have not written without taking much pains to enquire into the facts presented, and have abundant proof of what we write. We simply deem it a duty we owe our readers, to expose these abominable humbugs, that subsist by preying upon the unfortunate invalid, in common with other swindlers that have had reason to smart under our lash. When people learn not to trust any physicians but those of established reputation, and cease their migrations to every traveling charlatan that flaunts a flaming advertisement in their faces, then will there be no need of such articles as these; until then we shall continue to punch and kick every villainous swindler that presents himself to the public gaze.

